# Supplementary figures and images for: qHTSWaterfall: 3-dimensional visualization software for quantitative high-throughput screening (qHTS) data
Source: J Cheminform. 2023 Mar 31;15:39. doi: 10.1186/s13321-023-00717-9 (PMC10064508; doi:10.1186/s13321-023-00717-9)

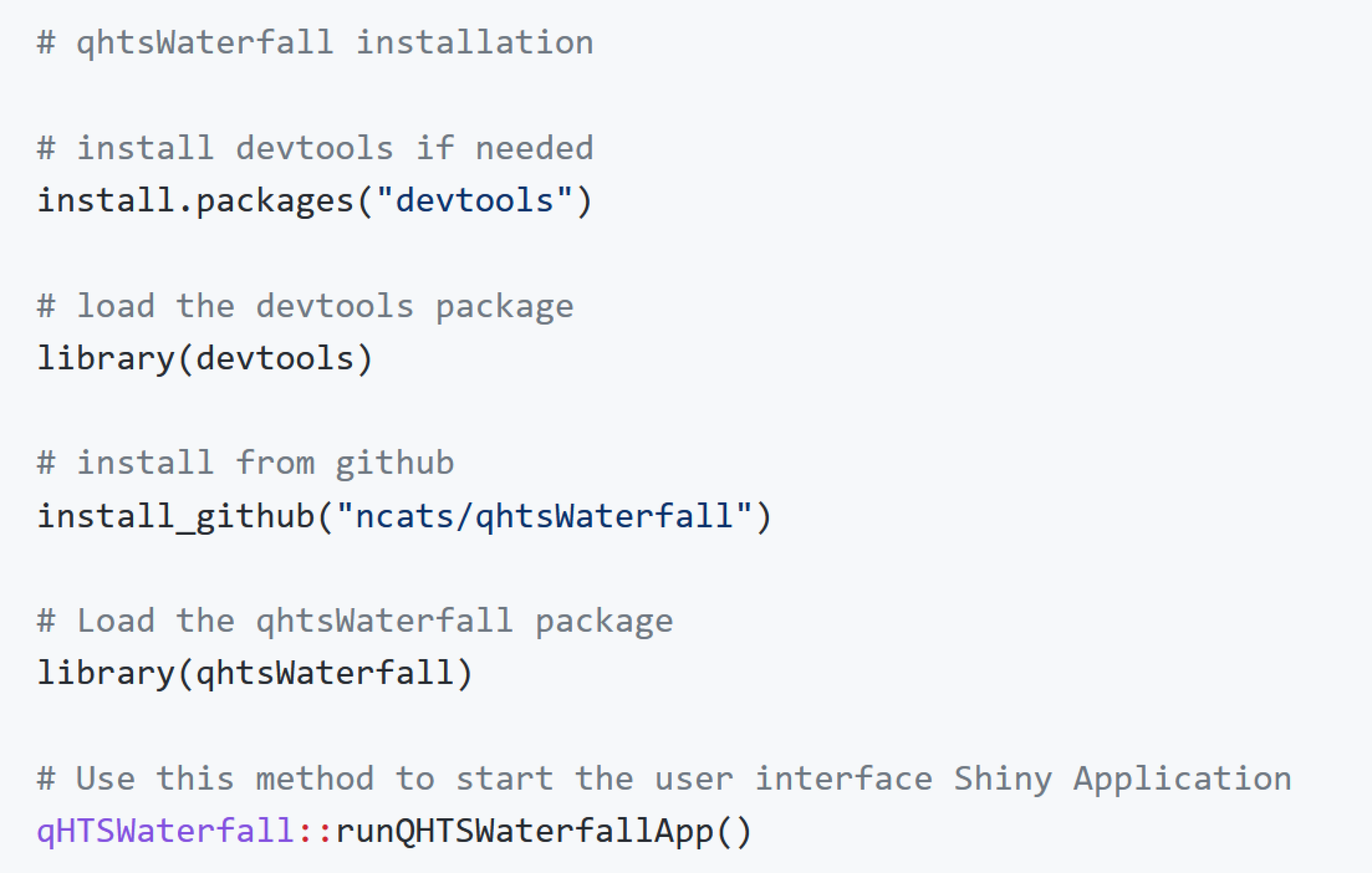

Supplement: Supplementary file 1 — Additional file 1: Fig. S1. Instructions for installation and starting the qHTSWaterfall Application. The package devtools is required for installation from github.com and can be installed if needed. [file 13321_2023_717_MOESM1_ESM.tif]

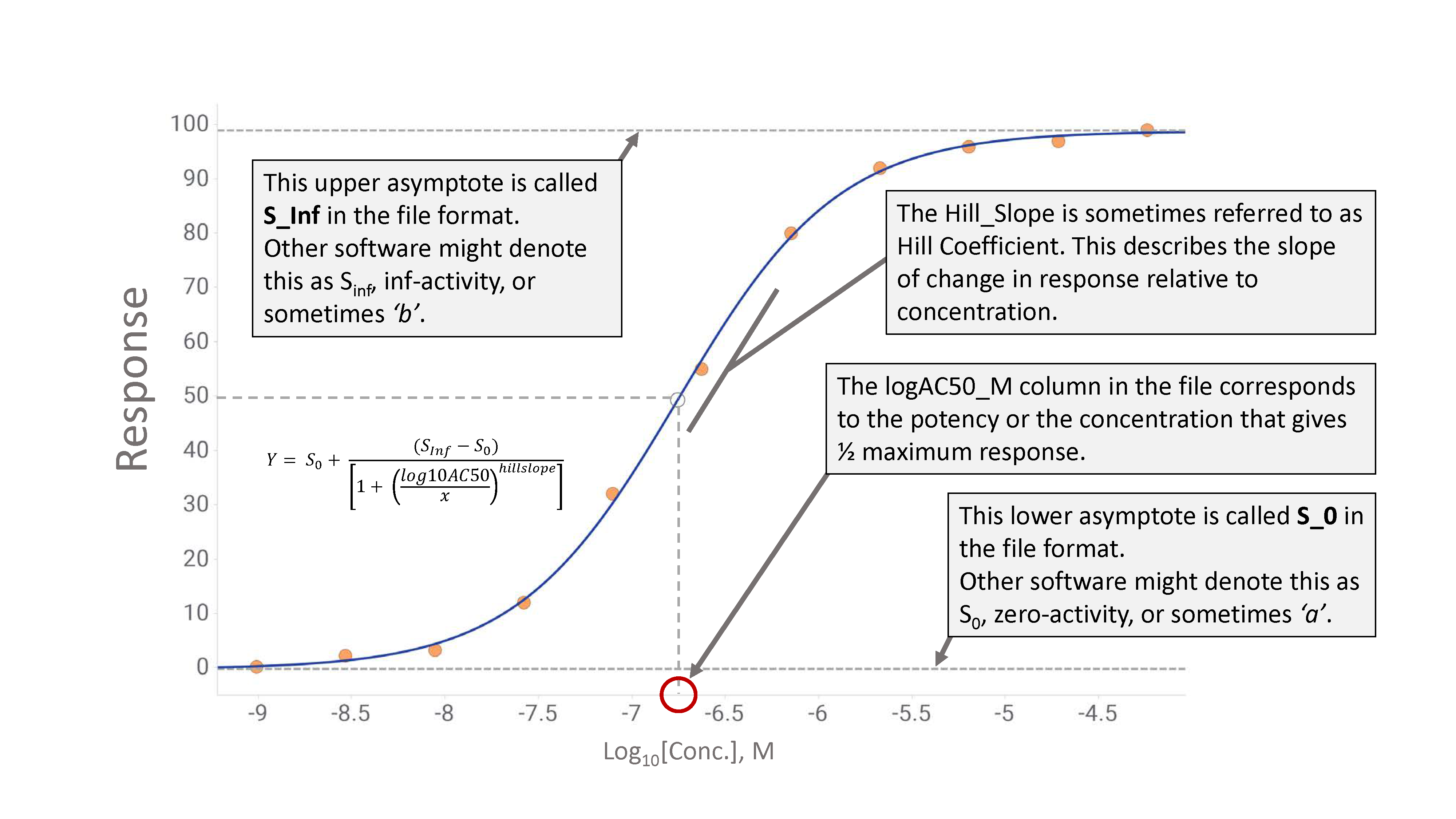

Supplement: Supplementary file 2 — Additional file 2: Fig. S2. A sigmoidal concentration response curve. The 4 parameters contained in the input file (denoted in the file as S_0, S_Inf, Hill_Slope and logAC50) are explained here. Note that some software programs that generate these fit parameters may use different nomenclature to refer to these parameters. [file 13321_2023_717_MOESM2_ESM.tif]
